# Molecular Mechanisms of Drosophila Hematopoiesis

**DOI:** 10.32607/actanaturae.27410

**Published:** 2024

**Authors:** S. A. Sinenko

**Affiliations:** Institute of Cytology Russian Academy of Sciences, St. Petersburg, 194064 Russian Federation

**Keywords:** hematopoiesis, hematopoietic organ, multipotency, hematopoietic stem cells, hematopoietic niche, Drosophila melanogaster, hemocytes, differentiation, signaling pathways, transcription factors

## Abstract

As a model organism, the fruit fly (*Drosophila melanogaster*) has assumed a leading position in modern biological research. The *Drosophila *genetic system has a number of advantages making it a key model in investigating the molecular mechanisms of metazoan developmental processes. Over the past two decades, significant progress has been made in understanding the molecular mechanisms regulating *Drosophila* hematopoiesis. This review discusses the major advances in investigating the molecular mechanisms involved in maintaining the population of multipotent progenitor cells and their differentiation into mature hemocytes in the hematopoietic organ of the *Drosophila *larva. The use of the *Drosophila *hematopoietic organ as a model system for hematopoiesis has allowed to characterize the complex interactions between signaling pathways and transcription factors in regulating the maintenance and differentiation of progenitor cells through the signals from the hematopoietic niche, autocrine and paracrine signals, and the signals emanated by differentiated cells.

## INTRODUCTION

The fruit fly (*Drosophila melanogaster*) is a model organism that has been widely used in genetic studies in cell biology, developmental biology, and immunology. It has been more than 100 years since Thomas Hunt Morgan began using this model system in genetic research [1, 2]. For genetic and biomedical research, *Drosophila *provided several advantages: (1) a minimal set of chromosomes of only four pairs, three of which (X/Y, II, III) virtually contain all the genes of the organism; (2) *Drosophila*’s fully sequenced and annotated genome consists of approximately 13,767 genes and is characterized by a minimum number of duplicated genes and minimal gene redundancy; (3) the methods for producing mutant *Drosophila *lines have been well developed and include chemical, isotope, transposon (P-element) and CRISPR/Cas9-mediated mutagenesis, UAS/Gal4- mediated conditional inactivation of gene expression through interfering RNA (RNAi) and ectopic gene expression, as well as lines with the visualized tissues of interest [3, 4, 5, 6, 7, 8, 9, 10, 11]. The targeted gene inactivation methods allow one to implement the reverse genetics approach involving inactivation of a gene of interest while investigating its phenotype/function in a living organism. The fruit fly is perfect for extensive genetic screens using the forward genetics approach as a means to identify mutations and gene function after detection of the phenotype of interest [12, 13, 14], and modified genetic screens aimed at identifying the genes involved in the process of interest [15, 16, 17]; (4) International repository centers preserve extensive collections of mutant *Drosophila *lines, including those with genetic deletions, point mutations and P-transposon, CRISPR/Cas9, promoter-Gal4, UAS-RNAi, and UAS-transgenes lines; (5) the fruit fly has a stable system for mutation maintenance, using balancing chromosomes and combining mutations through meiotic recombination; (6) it make feasible phenotype studies at the organismal level *in vivo*; and (7) The fly has a short life cycle (30 days), and the fly stocks are convenient and relatively inexpensive to store and maintain. The disadvantages of this popular model are (1) a huge evolutionary distance between insects and mammals and, as a consequence, insufficient homology at the genetic and physiological levels; (2) the fruit fly’s small size makes it labor-intensive to process *Drosophila *tissues; and (3) the model limits the application of biochemical and immunochemical methods.

As a model system, *Drosophila *has been intensively used over the last 50 years in almost all areas of modern biology, from deciphering the molecular mechanism of apoptosis to investigations of aging mechanisms [3, 18, 19, 20, 21, 22, 23]. It has also been widely used to investigate the molecular mechanisms of hematopoiesis and the humoral and cellular responses of innate immunity. The term hematopoiesis, meaning a process of blood cell formation, development, and maturation, has historically referred to the blood cells of vertebrates whose hematopoiesis is maintained by hematopoietic stem cells (HSCs), giving rise to a number of multipotent and restricted hematopoietic progenitors that differentiate into all types of blood cells such as red blood cells, platelets, leukocytes, and lymphocytes. In invertebrate coelomic organisms, to whom *Drosophila *belong, the internal body cavity contains coelomic fluid or hemolymph carrying hemocytes that are analogs of the blood cells of vertebrates [24, 25, 26, 27]. Hematopoiesis in *Drosophila *is a process of multipotent progenitor cells maintenance and differentiation into three types of mature hemocytes occurring in several parts during life-cycle stages. It is important to note that insect hemocytes are functionally homologous to the myeloid cells of vertebrate innate immunity, with which they have evolved in parallel [28].

Dipteran insects have four life cycle stages; namely embryonic, larval, pupal, and imago. The main biological functions of *Drosophila *hemocytes are defensive, including the nonspecific humoral and cellular immune responses, participation in regenerative processes, and scavenging dead cells during ontogenesis.* Drosophila *is known to have three lines of mature hemolymph cells or hemocytes. They are plasmatocytes (PL), crystal cells (CCs), and lamellocytes (LM). The larval instars are characterized by significant growth and morphogenetic changes in the organism, accompanied by active defense against pathogenic microorganisms. During this stage, which is widely used to study hematopoiesis, the process of hematopoiesis occurs in the hematopoietic organ (HO) in which the temporal and spatial dynamics of progenitor cells maintenance and differentiation into all types of mature hemocytes can be observed. Investigations of* Drosophila *hematopoiesis have shown that the mechanisms that help maintain the multipotent hemocyte precursors of the fruit fly and of mammalian HSCs present significant differences. The *Drosophila *hematopoietic system does not have (or has not yet been identified to possess) *bona fide *multipotent stem cells that are analogous to the hematopoietic stem cells of vertebrates, which are maintained throughout life. Employing the *Drosophila *genetic model system has allowed for significant advances in deciphering and understanding the molecular mechanisms of hematopoiesis. The studies performed over the last two decades have demonstrated that the molecular mechanisms to maintain progenitor cells and ensure their differentiation into various hemocyte lineages are somewhat analogous to the processes of myeloidcell differentiation regulation in mammals [27, 29]. To date, a number of comprehensive review papers have been published that cover many of the issues in this research field [27, 29, 30, 31, 32, 33, 34]. This review discusses the major advances in the study of the molecular mechanisms of hematopoiesis in the *Drosophila *HO; these include regulation of multipotent progenitor cell maintenance and their differentiation by transcription factors, signaling pathways, and metabolic and environmental factors.

## HEMATOPOIETIC SITES IN DROSOPHILA

**Fig. 1:**
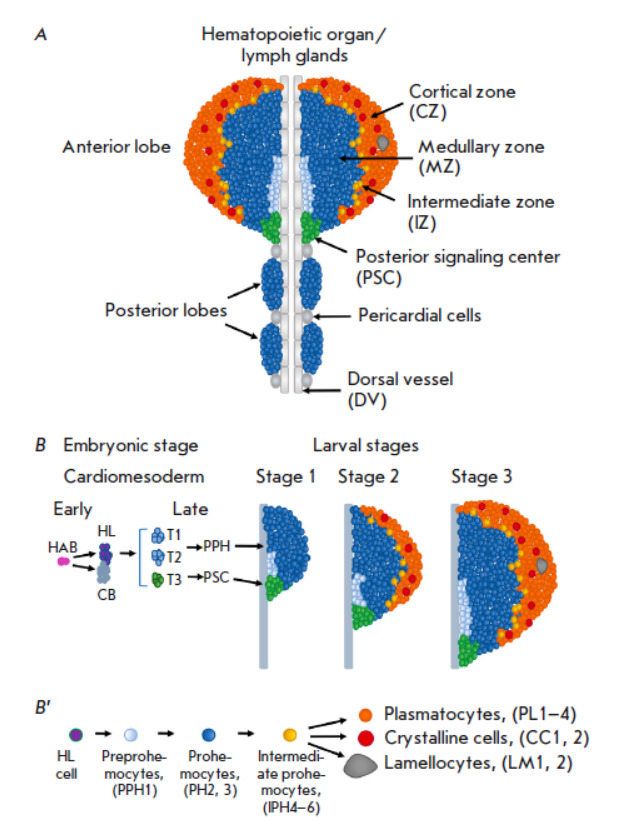
(*A*) *Drosophila *hematopoietic organ (lymph gland of the third larval instar) structure. The HO consists of paired anterior and posterior lobes, attached and interacting with DV and pericardial cells. The anterior lobes of HO are a model system for studying *Drosophila *hematopoiesis. They consist of cell populations or cellular zones of the PSC (hematopoietic niche); the medullary zone (MZ) involving preprohemocyte (PPH) and prohemocyte (PH) populations; the cortical zone (CZ), consisting of differentiated hemocytes such as plasmatocytes (PLs), crystal cells (CCs), and lamellocytes (LMs); and intermediate prohemocytes (IPHs) of the intermediate zone (IZ). (*B*) Genesis of hematopoietic organ. At the early embryonic stage, cardiogenic mesoderm cells or hemangioblasts (HAB) give rise to hematopoietic lineage (HL) and cardiovascular precursor cells, cardioblasts (CB). At subsequent embryonic stages, three pairs of thoracic segments (T1–3) of cardiogenic mesoderm produce the HO’s anterior pairs. Two anterior segments (T1–2) fuse and give rise to PPHs and all the hemocytes of the HO anterior lobe, while the third posterior segment produces PSC cells (highlighted in green). At the first instar larva, the anterior lobes contain PPH, PH, and PSC cells. At the second instar larva, PHs begin to differentiate into IPHs, which differentiate into plasmatocytes and CCs, forming the HO’s CZ (these hemocyte lineages are highlighted in colors, as shown on panel *B’*). At the third larval stage, IPH differentiation into terminally differentiated hemocytes continues, accompanied by CZ growth. At this stage, MZ PHs are maintained in a mitotically quiescent state. (*B`*) Hematopoiesis occurring in the HO. Hematopoietic progenitor cells and differentiated hemocyte lineages are indicated, and the abbreviations of the subtypes of the hemocyte lineages detected by scRNAseq are shown in parentheses

In *Drosophila*, origin and formation of early progenitor cells, or preprohemocytes (PPHs), occur in two independent (cephalic and dorsal) regions of the embryonic mesoderm. That means hematopoiesis in* Drosophila *occurs in two independent pathways or “waves”. In the first case, the cells of the cephalic mesoderm of the early embryo give rise to embryonic prohemocytes (PHs), which are further maintained and differentiate into the mature hemocytes that freely circulate in the hemolymph; hence they are named circulating hemocytes [35, 36, 37, 38]. PHs (and their derivatives) of this origin are maintained in the circulating hemolymph during all subsequent stages of the insect’s life cycle. The second wave occurs in the dorsal mesoderm, where the dorsal “blood” vessel, or “aorta” (DV, dorsal vessel), and the HO (originally named lymph gland) are formed (*Fig. 1*). However, the term HO is the most accurate for this organ [39, 40, 41]. This is a paired tissue formation consisting of hemocytes and their precursors bounded by an extracellular-matrix sheath. At the larval instars, the HO is the main site for maintaining PHs and differentiating them into mature hemocytes. During this stage, hemocytes do not leave the HO until the pupal stage begins. As for the circulating hemocytes, all their types are present in the hemolymph throughout the larval instars. When the pupal stage begins, the HO disintegrates and releases hemocytes, which then mix with the circulating ones. In this way, the PHs and the hemocytes originating from both sites of the mesoderm coexist during the postlarval stages of the fly’s life cycle [36, 38, 42, 43].

## DROSOPHILA HEMOCYTES

The mature hemocytes of the fruit fly are represented by three morphologically distinct types. These are plasmatocytes, phagocytic cells that perform defense, antimicrobial, and regulatory functions that comprise approximately 90–95% of hemocytes; crystal cells, non-phagocytic cells that make up 2–5% of hemocytes and are involved in wound healing, innate immunity reactions, and hypoxia; and lamellocytes, which are specialized giant cells that differentiate only in response to a parasitic organism invasion or upon tissue damage (*Fig. 1B`*). These cell types have been identified by ultrastructural studies and then confirmed by functional activity and molecular markers. Extensive studies have defined the signaling pathways and transcription factors that enable the specification, differentiation, and maintenance of these cell lines (see reviews [27, 29, 32]). Moreover, with single-cell RNA, sequencing (scRNAseq) has been detected in wide diversity in circulating hemocyte subgroups and eight of their subgroups with different functions have been identified using several experimental approaches [44, 45, 46, 47]. In the HO, previously undescribed cell types have also been identified, such as early precursors, or PPHs, and adipohemocytes, a PL subtype [43]. To date, however, many recently identified hemocyte subsets remain poorly characterized and their molecular and functional features require further study.

**Plasmatocytes**

PLs are the main type of *Drosophila *blood cells that perform defense, immune, and homeostatic functions. These cells are phagocytes that participate in the inactivation of pathogens and the scavenging of apoptotic cells during organism development [26, 48, 49, 50]. PLs perform phagocytosis via the Croquemort (Crq), Eater, and Nimrod C1 (NimC1) receptors [51, 52, 53, 54] and perform defense functions by secreting antimicrobial peptides (AMPs) (*Fig. 1B`*, *Table 1*) [55, 56, 57]. These cells secrete extracellular matrix (ECM) proteins, collagen IV, perlecan and laminin A, contributing to tissue formation [58, 59], and they synthesize peroxidasin (Pxn) [60], an enzyme meant to scavenge free radicals. PL ablation during embryogenesis engenders defects in organogenesis that lead to reduced embryo viability [61, 62, 63, 64]. ScRNAseq-based identification of molecular markers has allowed researchers to distinguish four PL subtypes (*Fig. 1B`*) [43].

**Crystal cells**

Crystal cells are characterized by the fact that they contain the crystals of propenoloxidases 1 and 2 (PPO1 and 2) that are involved in melanization. These cells participate in defense reactions upon tissue damage, as well as in the innate immune response, primarily through the activation of a biochemical melanization cascade [65, 66, 67, 68] that is functionally similar to the thrombosis cascade in mammals. Upon melanization, damaged tissues darken and harden, which is also associated with the production of reactive oxygen species (ROS) that participate in pathogen neutralization and healing of the damaged tissues (*Fig. 1B`*) [55, 65, 66, 69]. Suppressed melanization delays wound healing [70, 71, 72] and reduces susceptibility to microbial infections [65, 66]. CCs are unable to phagocyte; they express specific molecular markers and proliferate upon certain signals (see further, *Fig. 1B`*, *Table 1*). Using scRNAseq, two CC subtypes have been identified (CC1 and 2) [43].

**Lamellocytes**

Lamellocytes are large flat cells whose differentiation is induced by the signals from an invaded parasitic organism or an injury to tissue. The cellular immune response in *Drosophila *is mediated specifically by LMs and is mainly directed toward inactivating the eggs of parasitic wasps (*Leptopilina boulardi*) through their encapsulation [73, 74]. Plasmatocytes attach to the surface of an invading foreign object and then differentiate into LMs [75]. Mature LMs express specific molecular markers, and they are unable to divide or to phagocytize (*Fig. 1B`*, *Table 1*) [13, 26, 30, 51, 55, 66, 75, 76, 77, 78, 79, 80, 81]. Using scRNAseq, two LM subtypes have been identified in the HO (LM1 and 2) [43].

**Table 1:** Molecular markers and genes involved in the specification and maintenance of hemocyte lineages during *Drosophila* hematopoiesis

Drosophila HO cells	Hemocyte-type molecular markers	Human genes homologous to hemocyte marker genes	Genes and factors involved in hemocyte-type specification and maintenance	Human genes homologous to Drosophila one
Embryonic hemangioblasts	Odd-skipped (Odd) Serpent (Srp)	OSR2 GATA1	Odd Srp	OSR2 GATA1
PSC cells (embryonic T3 segment derivatives)	Antennapedia (Antp) Collier (Col) Hedgehog (Hh) Serrate (Ser) Wingless (Wg) Spitz (Spi) Pvf1	HOXA7 EBF1 SHH JAG1 WNT1 EPGN FLT1,4	Antp Col Wg Fz2 Myc Robo1,2 Dpp Dad Mad	HOXA7 EBF1 WNT1 FZD5 MYC ROBO1,2,3 BMP2 SMAD6 SMAD1
Hematopoietic lineage (embryonic T1–2 segment PPHs)	Homothorax (Hth)	MEIS1	Homothorax (Hth) Decapentaplegic (Dpp) Tinman (Tin) Pannier (Pnr) Heartless (Htl) Wingless/Wg	MEIS1 BMP2 NKX2-2 GATA4 FGFR3 WNT1
PPHs	Dome-/Pvf2 Notch-GAL4 Su(H)-lacZ E(spl)mβ Hand	VEGF A–D NOTCH1 RBPJ HES2 HAND1,2	Odd Pvf2/Pvr Notch Dpp Mad Scalloped (Sd)	OSR2 VEGF A–D NOTCH1 BMP2 SMAD1 TEAD1
PHs	Dome^+^ Е-cad Upd3 Wg	PTPRQ CELSR1 – WNT1	Patched (Ptc) Cubitus interruptus (Ci) Wg Wnt6 β-catenin Fz2 Col Stat92E AdoR Pka-C EGFR	PTCH1 GLI3 WNT1 WNT6 CTNNB1 FZD5 EBF1 STAT5A ADORA2A PRKACB EGFR
Intermediate PHs	Dome^+^ /Pxn^+^ Dome^+^ /Hml^+^		EGFR Pointed (Pnt)	EGFR ETS1
Plasmacytes	Peroxidasin (Pxn) Hemollectin (Hml) Nimrod (NimC) Eater Pvr	PXDN MUC5AC SCARF1 MEGF10 FLT1,4	Thisbe (Ths) Heartless (Htl) Pointed (Pnt) u-shaped (Ush) Srp FoxO Pvr	FGF8 FGFR3 ETS1 ZFPM1 GATA1 FOXO3 FLT1,4
CCs	Lozenge (Lz) Hindsight (Hnt) Sima/Hif-α Frizzled2 (Fz2) PPO1 and PPO2	RUNX1,3 RREB1 HIF1A FZD5 –	Notch Serrate (Ser) FoxO Fz2	NOTCH1 JAG1 FOXO3 FZD5
LMs	L1/Atilla Misshapen Myospheroid	– MINK1 ITGB1	EGFR FoxO Ph-p E(Pc) Col	EGFR FOXO3 PHC3 EPC1 EBF1

Note. Columns 3 and 5 indicate the human genes homologous to the corresponding Drosophila genes indicated in columns 2 and 4. The genes encoding the negative regulators of the corresponding hematopoiesis processes are marked in blue.

**The features of the stem cells of the *Drosophila* hematopoietic system: prohemocytes**

In mammals, HSCs are multipotent adult stem cells capable of self-renewing and differentiation into all blood cell types. They are maintained in a mitotically quiescent state, residing in the hematopoietic niches in bone marrow and other sites of hematopoiesis, where under certain external signals they undergo asymmetric division and further self-renewal and differentiation take place [82, 83, 84, 85]. HSCs are capable of repopulating the niches and replenishing the entire blood-cell repertoire. In *Drosophila*, the stem cells capable of self-renewal throughout life include the male and female germlines, intestinal, and neuronal stem cells [86, 87, 88, 89]. To date, no *bona fide* HSCs have been identified in the fruit fly, but early multipotent progenitor cells or preprohemocytes have been identified that are maintained by signals from the HO hematopoietic niche and DV cells. PPHs actively proliferate and give rise to more differentiated cells, namely prohemocytes. PHs are maintained in a mitotically quiescent state, and they are able to differentiate into all types of hemocytes [32, 38, 43, 90, 91]. It has not been established whether PPHs or PHs are capable of asymmetric division [92, 93] resulting in stem and differentiating daughter cells. The fact that *Drosophila*’s short life span frees it from the need to maintain and renew a large number of blood cells speaks in favor of its hematopoiesis mechanism being fundamentally different than that of HSC-based vertebrates.

## DROSOPHILA HEMATOPOIETIC ORGAN: ZONES, CELLS AND MARKERS

**Genesis of hematopoietic organ**

Clonal analysis has demonstrated that HO and DV progenitor cells are derived from a common progenitor cell, the so-called hemangioblast. These cells divide into two daughter cells, one of which is a precursor of cardiovascular cells (cardioblasts) that differentiate into DV cells, and the other is a precursor of the cells of the hematopoietic lineage that gives rise to hemocytes [94]. It is plausible that a similar mechanism exists in the hemangioblasts of the vertebral aorta–gonad–mesonephros (AGM) region that produces hematopoietic and vascular cells in vertebrates [95]. The HO is formed from the three thoracic segments (T1–T3) expressing the Odd-skipped (Odd) and GATA Serpent (Srp) TFs (*Fig. 1*, *Table 1*) [94]. At the same time, Antennapedia (Antp) TF induce and specify T3 cells to form the so-called posterior signaling center (PSC) consisting of about 30–40 cells (Fig. 1B) [96]. The PSC is a hematopoietic niche controlling hematopoiesis in the larval HO [97]. The Collier TF (Col), controlled by Antp [96, 97], also participates in PSC maintenance. T1–T2 segments form primary HO lobes through the activity of the Homothorax (Hth) transcriptional cofactor (*Fig. 1B*) [96]. The Tinman (Tin) and GATA Pannier (Pnr) TF genes, Decapentaplegic (Dpp) morphogen ligand, and Heartless (Htl) fibroblast growth factor receptor are required for HO cell formation. In addition, the Wnt/Wingless (Wnt/Wg) signaling pathway positively regulates cardiogenic mesoderm specification [94].

**Hematopoietic organ structure**

The fruit fly’s HO is a paired organ consisting of four lobes located along the aorta (*Fig. 1A*). The main lobe is the largest anterior or primary lobe of HO. In this lobe the coordinated processes PPH and PH maintenance and their proliferation and differentiation occur. The secondary, tertiary, and quaternary lobes are the least studied; they are several times smaller and serve as an additional source of hemocytes when a cellular immune response is activated [98]. The anterior lobe that is often called HO is the most structured part of the organ, so it has been used as a model or the main object to study the molecular mechanisms of hematopoiesis in *Drosophila *[41].

**Fig. 2:**
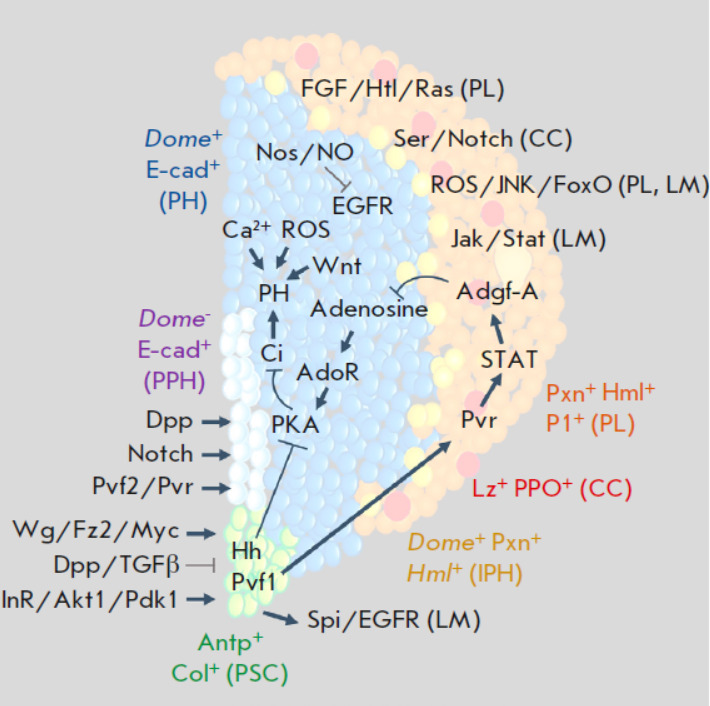
Schematic representation of the participation and interaction of the main signaling pathways and TFs in the regulation of HO hematopoiesis in the fruit fly. PSC-cell (in green) maintenance and proliferation are positively and negatively controlled by the respective Wg/Fz2/Myc and Dpp/TGFβ signalling pathways. PPH (in grey) maintenance and proliferation are positively controlled by the Dpp, Notch, and Pvf2/Pvr signals. PH (in blue) maintenance (PH, blue) is positively controlled by the Hh/PKA/Ci signals from the hematopoietic niche, autocrine signals Wnt/Fz/Fz2 and Ca^2+^ and negatively controlled by the Adgf-A signal originating from differentiated CZ hemocytes (in orange). PSC cells positively control Adgf-A expression through activation of Pvr and STAT in differentiated CZ hemocytes, being a link in the equilibrium signals between PSC cells and the mature hemocytes that control PH maintenance. IPHs are marked in yellow. PL differentiation and proliferation is positively regulated by FGF/Htl/Ras and ROS/JNK/FoxO: those of CC, by Ser/Notch; and those of LM, by Spi/EGFR, Jak/Stat, and ROS/JNK/FoxO (see details in the text)

Several zones are distinguished in the anterior lobe, each of them containing functionally different types of cells that are at different stages of differentiation: (1) the PSC that functions as a niche for regulating the self-renewal and differentiation of prohemocytes; (2) the medially located medullary zone (MZ), consisting of PPHs and PHs; (3) the distally located cortical zone (CZ) where differentiation and accumulation of mature hemocytes takes place (*Fig. 1*) [41]; and (4) the intermediate zone (IZ) located between the medullary and cortical zones containing intermediate PHs (IPHs) and expressing both PH and mature-hemocyte markers (*Fig. 1* and *Fig. 2*, *Table 1*) [43, 93, 99, 100].

As noted above, the PSC was the first zone to emerge as a distinct cell population. Its cells regulate PH maintenance and differentiation in the HO’s primary lobe throughout the larval stages. They perform only signaling functions and do not differentiate into hemocytes [43, 91, 96, 101, 102, 103, 104]. PSC cells express such molecular markers as Antp, Col, the Hedgehog (Hh) signaling pathway ligand, the Serrate receptor (Ser) ligand of the Notch (N) signaling pathway, and the Wg ligand of the Wg/Wnt signaling pathway (*Fig. 2*, *Table 1*) [96, 97, 99, 105].

Until the mid-second larval instar, only *Dome+ *prohemocytes expressing the *Domeless-Gal4 (Dome-Gal4)* reporter and a PPH population that does not express this reporter are present in the anterior lobe (see further). The *Dome+ *PHs are maintained at the second and third larval instar and differentiate into mature hemocytes forming the CZ [6, 41, 90, 91, 99, 106]. A given population of PHs is capable of self-renewal while producing mature hemocytes [90]. Clonal analysis has shown that *Drosophila *hematopoietic “stem” cells can be located in close proximity to the PSC [90, 91]. The presence of this cell population, referred to as PPHs, or PH1, was confirmed by scRNAseq [43]. However, as has been mentioned previously, the selfrenewal and asymmetric-division function characteristic of mammalian HSCs has not been identified in the hematopoietic “stem” cells of *Drosophila *[92, 93, 107]. At the first larval instar, *Dome*- PPHs are in direct contact with the dorsal aorta and the PSC. They are assumed to give rise to *Dome*+ PHs [41, 43, 90, 91] that actively grow and divide during the first and early second larval instar [41, 91]. ScRNAseq has shown that *Dome+ *PHs are a heterogeneous population consisting of two cell subtypes (PH 2,3), likely reflecting their differentiation hierarchy [43].

*Dome+ *PH proliferation significantly decreases by the middle of the second larval instar. At the same time, cells at the distal edge of the MZ begin to differentiate, which is accompanied by decreased proliferation, increased granularity, and the absence of E-cadherin (E-cad) expression. MZ cells, or *Dome+* PHs, are characterized by high expression levels of Upd3 (JAK/STAT signaling pathway) and Wg ligands, E-cad and ROS [41, 99, 100, 108], and a low Col expression level [102, 109, 110]. ECM proteins, including type-IV collagen (Viking, Vkg) and Trol perlecan, have preferred localization between MZ cells [106, 111].

In the CZ, plasmatocytes express the following markers: Pxn, Hemollectin (Hml), Eater, and the P1 antigen or Nimrod-C (NimC) (*Fig. 1B’*, *Table 1*) [41, 56, 81, 99, 112, 113]. ScRNAseq has identified four plasmatocytes subtypes [43]. CCs express such transcription factors as Lozenge (Lz), Hindsight (Hnt), Sima/Hif-α, the Frizzled2 (Fz2) receptor, PPO1, and PPO_2_ [65, 78, 114, 115, 116]. In the absence of exposure to pathogenic factors, LMs hardly ever form in the CZ. Two LM subtypes are differentiated in the HO, expressing L1/Atilla, Misshapen, and integrin α-PS4 and its partner Myospheroid (*Fig. 1B`*, *Table 1*) [10, 43, 51, 55, 77, 80, 117].

Between *Dome+ *PHs and differentiating Pxn+ cells of the medullary and cortical zones reside a population of cells that simultaneously express markers of both zones. These are the so-called intermediate prohemocytes (IPHs) that represent the IZ (*Fig. 1*, *Table 1*) [34, 93, 99, 100, 118]. IPHs express the early differentiation markers Hml and Pxn, but they do not express the mature plasmatocytes marker (P1) and CCs marker (PPO1 and 2) [106]. They also cease to express E-cad. Recent scRNAseq studies have allowed for a more detailed characterization of this zone, which includes four stages of IPHs (PH4–6), early plasmatocytes (PL1), and early CCs (CC1) [43]. It has also been demonstrated that IPH cells are characterized by mitosis activation and differentiate into plasmatocytes and CCs if activated by the Ras/Raf or Ser/Notch signaling pathways, respectively [118]. The molecular mechanisms regulating this population are the least understood and require further investigations.

## SIGNALING PATHWAYS INVOLVED IN MAINTENANCE AND DIFFERENTIATION OF PREPROHEMOCYTES

At the beginning of the first larval stage, the HO contains a population of multipotent PPHs, representing the earliest postembryonic population of hematopoietic progenitors that most likely disappear later than the first larval instar [91]. These cells are characterized by the lack of *Dome *PH marker expression, a low level of Dorothy (Dot)-marker expression, the activated Notch signaling pathway (*Notch-GAL4, Su(H)-lacZ*) and its target gene *enhancer of split mβ (E(spl)mβ)* (*Fig. 1*, *Table 1*) [43, 91]. In addition to Notch, maintenance of these cells is regulated by the Dpp ligand secreted by PSC cells. Dpp inactivation in the PSC or suppression of the mothers against the dpp (Mad) function in Notch+ PPHs causes a significant reduction in the HO size by the 3^rd^ larval instar. In other words, activation of the Notch and Dpp signaling pathways is required for PPH proliferation. During the 2^md^ and 3^rd^ larval stages, *Dome- *PPHs begin to express the Hand and Scalloped (Sd) TFs [119]. These cells have been found to express the Pvf2 ligand of the Pvr receptor (human PDGF/VEGF receptor homolog), and its expression is dependent on Sd activity. Pvf2 inactivation in these cells leads to a suppression of their proliferation and a significant subsequent reduction in HO size. At the same time, ectopic Pvf2 expression in these cells restores the proliferative defect in the HOs that have experienced partial loss of the Sd function [119].

The calcium/calmodulin signaling pathway activated through the ionotropic γ-aminobutyric acid receptor (GABABR) has also been shown to be involved in the maintenance of early *Dome*+ PHs. GABABR is expressed in the PSC cells where the calcium/calmodulin pathway participates in the regulation of PPH proliferation at early larval stages without affecting hemocyte differentiation at the third larval instar. The disruption of the calcium/ calmodulin pathway in PSC causes a significant decrease in PPH proliferation [120]. These data indicate there are several signaling pathways involved in the maintenance and proliferation of early* Dome*- PPHs, in particular Notch, Dpp, and Pvf2/Pvr (*Fig. 2*). The involvement of several pathways in the regulation of one process may be indicative of a complex regulation system and a possibility of mutual compensation. It should be noted that the technical difficulties in working with the HO at the first larval instar and the lack of markers make it difficult to study the *Dome-Sd+ *and *Dome-Notch+ *cell populations [91].

## SIGNALING PATHWAYS INVOLVED IN MAINTENANCE AND DIFFERENTIATION PROHEMOCYTES

As previously mentioned, PHs are multipotent precursors of all hemocyte types. The multipotency and mitotic quiescence of these PHs is maintained through a variety of signals that come from three different sources (*Fig. 2*). The first type is the signals of the cytokines and growth factors secreted by the hematopoietic niche cells of the PSC. The second type is the autocrine or paracrine signals produced and received by the same population of cells in the HO’s MZ. The third type is signals from differentiated cells in the CZ that are controlled by the maintaining and differentiating MZ PHs. The additional fourth type includes systemic signals originating from various tissues outside the HO that are mediated mainly through PSC in response to environmental factors.

A characteristic feature of PHs is a strictly coordinated control of their proliferation. At the first and early second larval instar, practically all HO cells, excluding PPHs and PSC cells, are *Dome+* PHs (*Fig. 1B*). At these stages, prohemocytes intensively and asynchronously proliferate. Then, when differentiated cells begin to appear at the late phase of the second larval instar, PH proliferation slows down abruptly. Further, during CZ formation,* Dome+ *PHs practically cease to proliferate, while IZ and CZ cells continue at a higher proliferation rate throughout the entire third larval instar [41]. Therefore, a low proliferation rate and control over it correlate with maintenance of the prohemocyte multipotent state. As already mentioned, the four types of signals are necessary to maintain PHs in* Drosophila*: autocrine signals and signals coming from the PSC, differentiating cells, as well as signals from other tissues of the organism. Absence of any of these signals leads to the loss of PH multipotency and causes their proliferation and, consequently, differentiation [96, 103]. An important feature of PHs is correlation of their proliferation with an ability to differentiate. To date, a growing body of evidence seems to suggest that only proliferating PHs are able to accept differentiation signals, while resting PHs do not perceive them. Investigating the mechanisms regulation of the proliferative activity of intermediate PHs should contribute to a better understanding of this issue.

## PSC SIGNALS REGULATE MAINTENANCE AND DIFFERENTIATION OF PROHEMOCYTES

**Central role of the Hh/Ptc/Ci signaling pathway in maintaining a PH multipotent state**

PSC cells act as a hematopoietic niche in the HO to secrete a number of signaling ligands or growth factors while they do not express corresponding receptors. At the same time, the receptors of these ligands are expressed in prohemocytes and the inactivation of corresponding ligands in PSC cells inhibits prohemocyte maintenance and causes them to differentiate.

The Hh ligand binding to its receptor Patched (Ptc) activates TF Cubitus interruptus (Ci). Hh is expressed exclusively in PSC cells during the second and third larval instar (*Fig. 1*, *Table 1*). While Ptc and activated Ci are expressed at a high level in *Dome+ *prohemocytes, Hh inactivation does not affect PSC cells, but it stimulates the differentiation of *Dome+ *PHs to differentiate into all three types of hemocytes [96, 97, 102, 103, 104, 121, 122, 123, 124]. Besides, a suppressed Ci function causes PH differentiation, similar to the Hh inactivation in PSC cells (*Fig. 2*) [96, 121]. This process is enabled, among other things, due to the morphological features of PSC cells, whose prolong extended pseudopodia pass through several PH layers and allows delivery of the ligand deep inside the MZ [96, 102]. It has also been shown that PSC-cell ablation by apoptosisinduction does not cause the expected prohemocyte differentiation observed with the Hh inactivation [109, 110, 121]. However, it has been found that the* Dome+ *PH population is heterogeneous. As such, a portion of the cells (Odd+ Col-) respond to the Hh signal, whereas Odd+ Col+ cells are not sensitive to it [110, 121]. In this regard, PSC-cell ablation is assumed not to affect certain prohemocytes. It is possible that Col+ cells are a separate PH population that is controlled by signals from DV cells [91, 119]. It has also been found that the DV serves as an additional niche. Thus, the Branchless (Bnl) ligand (homologous to the fibroblast growth factor, FGF) produced by DV cells activates the FGF signaling pathway in PHs. When activated, it regulates the level of the intracellular calcium and contributes to PH maintenance in an undifferentiated state [125].

A suppressed *Roundabout *(*Robo*) gene function increases the number of PSC cells, and it also causes them to spread deeper into the HO. These events correlate with decreasing PL and CC differentiation [126]. At the same time, in response to a pathogenic invasion, the activity of the NF-kappaB Relish factor of the Imd signaling pathway is suppressed in PSC cells. The Relish inactivation manifests in the disruption of the PSC-cell cytoskeleton due to Jun-kinase activation, which leads to Hh-ligand retention thus disrupting the prohemocyte maintenance and causing their premature differentiation and activation of the cellular immune response [127]. It has also been shown that suppression of Ca^2+^ signaling or disruption of intercellular contacts between PSC cells affects their function and causes premature PH differentiation [128].

**ROS regulate lamellocyte differentiation through the activation of the Spitz/EGFR and Toll/Dif signaling pathways in PSC cells**

In addition to hematopoiesis regulation in the HO, PSC cells regulate lamellocyte differentiation inside and outside the HO. In this way, PSC-cell ablation through Col inactivation or apoptosis induction prevents differentiation of lamellocytes in response to a parasitic wasp infestation [97, 109]. Genetic methods have proved that this infestation leads to a significant increase in the ROS level in the PSC and that ROS are the key signal that induces lamellocyte differentiation [129]. ROS are not normally detected in PSC cells, but their level sharply increases when infected by parasitic wasps. Artificial increase in the ROS level in PSC cells due to the suppression of the mitochondrial respiratory chain also leads to a large-scale increase in the number of lamellocytes in the circulation and HO [129]. In both cases, ROS removal by mitochondrial superoxide dismutase 2 (SOD2) or catalase suppresses LM formation in the HO and circulation. In addition, activation of the Akt kinase signaling pathway (Akt1)/FoxO in PSC cells enhances the antioxidant response that abolishes LM generation. ROS have been shown to activate the epidermal growth factor receptor (EGFR) signaling pathway, enabling lamellocyte differentiation. Inactivation of Spitz (EGFR ligand) in PSC cells or the EGFR function in hemocytes suppresses LM formation (*Fig. 2*) [129]. The functions of the Star and Rhomboid proteins directly involved in the transport, cleavage, and activation of the Spitz ligand (its conversion into a soluble form), are necessary for LM induction. In addition, high ROS levels activate the Toll signaling pathway in PSC cells, which also contributes to LM induction in response to a parasitic wasp infestation [130]. Loss of the Toll signaling pathway components through inactivation of Dif and pelle disrupts LM formation. Along with many questions about the nature of ROS generation in PSC cells and signaling in response to a parasitic invasion, the question of interaction of the Spitz/EGFR and Toll/Dif signaling pathways in PSC cells in the regulation of LM differentiation remains unresolved.

## LOCAL SIGNALS TO SUPPORT THE MULTIPOTENT PROPERTIES OF PROHEMOCYTES

**Wg/Wnt/β-catenin signaling pathway**

One of the important pathways involved in the maintenance of multipotency and self-renewal of mammalian hematopoietic stem cells is the Wnt/β- catenin signaling pathway. The Wnt ligand signals act in the both autocrine and paracrine ways. In the latter case, ligands are secreted from hematopoietic niche cells and contribute to HSC identity maintenance. In *Drosophila*, as in mammals, several genes encoding the Wnt ligands (Wg, Wnt-2, -3/5, -4, -6, -8, -10) and two genes encoding their receptors Fz and Fz2 are known. Ligands binding to the receptors cause activation of either the canonical pathway through the activation of β-catenin TF (Armadillo, Arm) or the non-canonical pla - nar cell polarity signaling pathway, which activates transcription via JNK. The canonical Wg/Wnt/β- catenin signaling pathway is involved in the maintenance of the PH multipotent state (*Fig. 2*, *Table 1*) [99]. The Fz2 receptor that transduces signaling through the canonical pathway is expressed at a high level in *Dome+ *PHs. Enhanced activation of the Wg/Wnt/β-catenin signaling pathway in *Dome+* PHs due to the overexpression of the Wg ligand or the constitutively active form of β-catenin prevents these cells from differentiating and stimulating their maintenance in an undifferentiated state [99]. In turn, inhibition of this signaling pathway using a combination of dominant-negative forms of the Fz and Fz2 receptors in *Dome+ *PHs causes disruption of HO zonation; i.e., clusters of differentiated cells “intermingle” with PH clusters (*Fig. 2*). Simultaneous expression of the dominant-negative forms of Fz and Fz2 increases the number of intermediate prohemocytes [99]. This suppresses E-cad expression, a protein that is directly involved in PH maintenance. E-cad suppression in PHs causes their differentiation, while E-cad overexpression promotes PH maintenance [41, 131]. Activation of the Wg/Wnt/β-catenin signaling pathway in *Hml+* cells of the CZ has also been shown to suppress the expression of the Tig ECM protein and affect plasmatocyte maturation [132, 133], which is additional indication of the function of this signaling pathway in the IZ cells. Recent studies have demonstrated that the Wnt6 ligand, whose expression is controlled by the Hh signaling pathway, is also expressed in prohemocytes [134]. It is important to note that Wnt6 transmits signals through the new noncanonical Wnt-pathway mediated by the LRP6 receptor and suppressing β-catenin activity. The interaction of cytosolic β-catenin with E-cadherin suppresses the EGFR signaling pathway in PHs. Therefore, activation of the Wnt6/LRP6 pathway leads to cell cycle delay in the G2 phase, thus preventing prohemocytes from responding to signals for differentiation [134]. However, activation of the EGFR signaling pathway in intermediate prohemocytes of the IZ relieves cell cycle blockade by activating beta-catenin and allows cells to differentiate through Pointed (Pnt) TF activation [134]. Thus, activation of the signaling pathways – canonical Wg/ beta-catenin and non-canonical Wnt6 – is important for maintaining PHs in a multipotent state, possibly in different PH populations, including that of IZ intermediate prohemocytes.

**Calcium/calmodulin signaling pathway**

The calcium/calmodulin signaling pathway is involved not only in the PSC-dependent regulation of preprohemocyte proliferation, but also in the maintenance of *Dome+ *PHs (*Fig. 2*). Suppression of calcium signaling in prohemocytes leads to an increase in the number of differentiated hemocytes. On the contrary, activation of calcium signaling in PHs promotes their maintenance and proliferation, reducing significantly the number of formed mature hemocytes [120].

**Collier factor activity**

The Col TF is expressed in *Dome+* PHs, and its inactivation in these cells leads to their differentiation into plasmatocytes and CCs (*Table 1*) [109, 110]. The expression of this transcription factor in PHs is not controlled by signals from the PSC. At the same time, Col negatively regulates lamellocyte differentiation as well. A decrease in the level of Col has been observed during enhanced lamellocyte differentiation, while its ectopic expression in PHs prevents the formation of these cells. It remains unclear which signaling pathway activates the Col function in prohemocytes.

**The FGF and Gbb/TGF-beta signaling pathways**

Unlike Wnt, activation of the FGF signaling pathway in *Dome+ *PHs differentiates them into mature hemocytes of all three types. Inhibition of the FGF signaling pathway causes a significant prohemocyte growth and the suppression of their differentiation. Interestingly, FGF ligand Thisbe (Ths) and the Htl receptor are expressed in PHs and in some, probably, IPHs expressing peroxidasin. Ectopic expression of the FGF-targeted transcription factors Pnt and Ush promotes prohemocyte differentiation [135]. Therefore, FGF signaling through Htl, Ras/MAPK, Pnt, and Ush promotes prohemocyte differentiation (*Fig. 2*). It has also been shown that the TGF-beta signaling pathway, through the Glass bottom boat (Gbb) ligand, is involved in the negative regulation of lamellocyte and plasmatocyte differentiation in the CZ through the suppression of the EGFR and JNK signaling pathways [136].

**JAK/STAT signaling pathway**

The Unpaired 1–3 (Upd1–3) cytokines acting through the Dome receptor activate the JAK kinase and Stat92E TF, inducing the transcription of target genes [102, 137]. It has been shown that the JAK/STAT signaling pathway is activated in *Dome+* prohemocytes to maintain their identity and prevent differentiation [41, 119, 137]. The Stat92E TF activity in PHs is much lower than that in differentiated CZ hemocytes [138]. However, the Stat92E TF function is essential for PH maintenance. Stat92E inactivation by a temperature-sensitive mutation leads to PH differentiation [102]. At the same time, the inactivation of JAK/STAT signaling pathway components such as Dome or JAK kinase (hopscotch, hop), or Stat92E in MZ prohemocytes, does not affect their maintenance [103, 139]. The Ush TF regulated by JAK/STAT signaling has been shown to promote the expression of E-cad and Ptc in PHs, thus participating in their maintenance and differentiation suppression [131, 140]. The Asrij (Arj) protein is involved in the phosphorylation and activation of STAT. Arj inactivation partially phenocopies the temperature-sensitive Stat92E allele that suppresses PH maintenance and induces their differentiation [141, 142]. In addition, the JAK/STAT signaling pathway positively regulates PH differentiation into lamellocytes upon cellular immune response induction (*Fig. 2*, *Table 1*) [137].

**ROS are involved in the maintenance of prohemocytes**

The main ROS sources in the cell are the mitochondrial respiratory chain and membrane NADPH-oxidases (NOX). They generate superoxide anion radicals which then are converted into hydrogen peroxide by superoxide dismutases. The main cellular ROS forms are hydrogen peroxide and a superoxide anion radical. ROS are powerful oxidizing agents, so upon their high concentrations and when a cellular antioxidant system is disturbed, they cause irreversible changes in macromolecules, provoking cell aging and death. However, sublethal and physiological ROS concentrations serve as important signaling mediators involved in posttranslational modifications of signaling pathway proteins and transcription factors, thereby regulating various processes in the cell [143, 144]. Unexpectedly, it has turned out that increased ROS levels are normally maintained in the *Dome+ *PHs, being in mitotic quiescence if compared to differentiated CZ hemocytes (*Fig. 2*, *Table 1*) [100]. By analogy with quiescent mammalian HSCs, it can be assumed that these cells have low mitochondrial/respiratory activity and, consequently, low ROS levels. At the same time, mammalian myeloid precursors are known to have significantly higher ROS levels than that in HSCs, which also increases during differentiation of myeloid lineage cells. The mechanism used to generate increased ROS levels in prohemocytes remains unclear. ROS have been shown to function as signaling molecules during prohemocyte differentiation. Expression of antioxidant enzymes reduces the basal ROS level in *Dome+ *PHs and suppresses mature hemocytes formation. At the same time, induction of ROS excess and oxidative phosphorylation attenuation by the inactivation of mitochondrial respiratory chain complex I through the JNK signaling pathway in prohemocytes lead to their differentiation into the three types of mature hemocytes [100]. The increased ROS level in PHs also leads to a decrease in E-cad expression through the activated JNK signaling pathway and TF Srp [145]. Ectopic expression of the FoxO TFs of the JNK pathway in PHs causes their differentiation into plasmatocytes and crystal cells [100, 145]. Simultaneous FoxO activation and inactivation of the chromatin proteins Polyhomeotic proximal (Ph-p) and the Enhancer of polycomb (E(Pc)) causes PH differentiation into lamellocytes (*Fig. 2*, *Table 1*). Therefore, a moderately high but physiologically controlled ROS level is necessary for PH maintenance. However, increased production of mitochondrial ROS in PHs causes their differentiation by activation of the JNK/FoxO signaling pathway. It is noteworthy that in this context the FoxO function does not mediate antioxidant genes regulation. It has also been found that the putative PHs of the *Drosophila *larval circulation outside the HO produce high levels of ROS. These PHs have not yet been well characterized and are referred to as progenitors by analogy with the HO prohemocytes expressing increased levels of ROS and the Wg ligand [17]. These cells are generated in large excess due to the activity of the oncogenic chimeric AML1-ETO protein forcefully expressed in *Hml+* hemocytes. High ROS levels in such circulating PHs contribute to their maintenance and increased proliferation. The ectopic expression of the antioxidant enzyme SOD2 or catalase (Catalase, Cat), as well as of FoxO that activates their expression, is able to suppress the generation and excessive proliferation of hemocytes and their progenitors, all caused by the AML1-ETO oncogene [17]. In this case, it is most likely that the Akt1/FoxO signaling pathway canonically regulates antioxidant genes expression. Thus, similarities and significant differences can be observed in the regulation of the maintenance of the HO PHs and circulating PHs by ROS.

It has recently been shown that nitric oxide synthase (Nos) is particularly expressed in prohemocytes and that, through the production of nitric oxide (NO), it is involved in the posttranslational S-nitrosylation of the proteins on cysteine residues [146]. S-nitrosylation of proteins, together with cytosolic calcium, activates the Ire1-Xbp1-mediated unfolded protein response (UPR) necessary to maintain PHs in a mitotically inactive state by maintaining them in the G2 phase of the cell cycle [146]. As already mentioned, such a cell cycle block makes prohemocytes refractory to the paracrine factors inducing differentiation. It has also been shown that EGFR S-nitrosylation temporarily inactivates this receptor and, thus, renders the PHs unresponsive to the relevant signals. It is important to note that the Nos expressed in prohemocytes does not contain a reductase domain but is capable of generating NO [146]. In turn, since these cells have high ROS levels, this form of Nos can utilize ROS to synthesize NO. For that reason, it has been suggested that the interaction between ROS and NO may participate in the maintenance of appropriate levels of ROS by generating NO, and, thereby, protecting PHs from excessive ROS production.

In general, it has become evident that there is a complex network that regulates PH maintenance and differentiation in the HO and that involves several signaling pathways for local regulation of these processes. At the same time, there might be complex network interactions between the components of these signaling pathways in certain time intervals of *Drosophila *hematopoiesis. Different signaling pathways are able to induce cell differentiation, which may be indicative of the increased plasticity of *Drosophila *hematopoietic progenitor cells. Apart from these signals and the signals from the PSC, prohemocyte maintenance is controlled by signals from differentiated cells. This will be discussed in the next section.

## EQUILIBRIUM SIGNALS BETWEEN PSC CELLS AND MATURE CORTICAL HEMOCYTES REGULATE PROHEMOCYTE MAINTENANCE

The *Drosophila *genetic system has been used to identify a unique mechanism that regulates progenitor- cell maintenance. It has been found that prohemocyte maintenance and differentiation are controlled “in equilibrium” by two mechanisms: (1) directly by a signal from PSC cells; and (2) by the signal of differentiated daughter cells, which is also controlled by an additional signal originating from the same hematopoietic niche. PSC cells regulate not only the maintenance of the PH multipotent state, but also the maintenance and differentiation of CZ hemocytes (*Fig. 2*). This process is regulated by the Pvf1/Pvr signaling pathway [103]. The Pvf1 ligand is secreted in PSC cells, while the Pvr receptor is expressed at high levels in cortical-zone cells. Inactivation of Pvf1 in PSC cells does not affect their proliferation and number, but it suppresses PH maintenance, causing their differentiation. A similar effect is observed when the Pvr receptor function is suppressed in differentiated hemocytes of the CZ, causing extensive PH differentiation [103, 119]. It is important that the Pvf1 ligand is transported for long distances across multiple cells by transport vesicles that include bound-but-not-signaling complexes of Pvf1 and Pvr on the prohemocyte plasma membrane.

With the use of genetic methods it has been demonstrated that Pvf1, when interacting with Pvr of cortical hemocytes, activates the STATdependent expression of secreted adenosine deaminase of growth factor-A (Adgf-A) (*Fig. 2*). This enzyme deaminates adenosine, converting the extracellular signaling molecules of adenosine into inert inosine [147, 148]. Deletion of adenosine by Adgf-A in CZ hemocytes leads to the suppression of the corresponding signaling pathway through the adenosine receptor (AdoR) located in PHs. As a result, the activity of cAMP-dependent protein kinase A (PKA) is reduced, which, in turn, activates the transcription factor Ci that mediates the PH maintenance in a multipotent state. It is important to note that the activation of the Hh/Ptc signaling pathway from the PSC also inhibits PKA activity in PHs, which leads to Ci activation. Therefore, the Hh-dependent signal from PSC cells and the adenosine signal from differentiated CZ hemocytes synergistically inhibit PKA activity and activate Ci, promoting prohemocyte maintenance in the MZ [96, 103]. These data could be a sign that a similar equilibrium signal may also operate in the mammalian hematopoietic system.

## SIGNALING PATHWAYS MAINTAINING THE PSC-CELL FUNCTION

The Antp and Col TFs are expressed in PSC cells throughout all larval instar. These cells proliferate during the early larval instar and form a cluster of 30–40 cells that is maintained during the third larval instar (*Fig. 1*). Antp directly controls the specification, maintenance, and growth of these cells and activates the expression of Col, which in turn is involved in the maintenance of Antp expression [96, 97, 116]. The Serrate ligand of the Notch receptor is expressed later in a certain population of PSC cells and is required for CC differentiation in the CZ [96, 97, 105]. Two signaling pathways, Wg and Dpp, antagonistically regulate PSC-cell proliferation [99, 123]. All components of the Wg signaling pathway, Fz2, β-catenin/Arm, and Disheveled (Dsh) are expressed in the PSC. Wg activation is necessary to increase the number of PSC cells (*Fig. 2*, *Table 1*). Blocking the Fz2 function significantly decreases the number of PSC cells, while the ectopic expression of Wg leads to a significant increase in their number [99]. In contrast to Wg, suppression of the Dpp/TGF-beta signaling pathway increases the number of PSC cells [123]. Activation of the TGFbeta signaling pathway through the Dpp ligand ectopic expression activates the Daughters against the dpp (Dad) and Mad TFs expressed in PSC cells [123, 149]. The number of PSC cells significantly increases when this pathway is suppressed through inactivation of the Dally like (Dlp) heparan sulfate-proteoglycan-binding protein and pMad in these cells (*Fig. 2*). Simultaneous suppression of the Wg and Dpp signaling pathways restores the PSC to its wild-type size. The regulation of the number of PSC cells by Wg is Myc-dependent, since Myc inactivation reverses the increase in PSC cells caused by ectopic Wg expression [123]. In its turn, the Jumu TF of the fork head family is involved in Myc regulation while the last regulates PSC-cell proliferation [150]. Further studies are required for a detailed understanding of how these signaling pathways interact for the regulation of PSC-cell proliferation and functioning.

Studies have shown that the developed network of extracellular matrix proteins between PSC cells and PHs is important for the regulation of Dpp and Wg signaling during hematopoiesis in HO andin response to stress [151]. It has been found that the septated contacts between PSC cells are destroyed upon activation of the Toll or Imd signaling pathways or in response to a bacterial infection. Usually, the PSC-cell cluster is impermeable to large-molecule dyes. However, inactivation of the dense septated intercellular contact proteins Coracle (Cora) or Neurexin IV (Neurexin IV, NrxIV) leads to PSC-cell permeabilization. The increased permeability increases the number of PSC cells, decreases that of PHs, and promotes plasmatocyte and crystal cell differentiation. Losing such a barrier impairs Wg and Dpp ligand signaling [151] both within the niche and signaling to PHs. It has been shown that gap junctions (GJ) and the Ca^2+^-signaling pathway are involved in the regulation of Hh secretion [128].

In addition, the signals from the DV cells adjacent to the PSC have been proven to regulate proliferation, function, and localization of PSC cells. So, the glycoprotein Slit is secreted in the DV cells, whose receptors Roundabout 1 and 2 (Robo 1 and 2) are expressed on PSC cells. The interaction of Slit with Robo 1 and 2 regulates PSC-cell proliferation and localization [33, 126, 152]. Suppression of the Robo function in PSC or Slit expression in DV cells increases the number of PSC cells and causes them to expand deep into the HO, including through suppressed E-cad expression [126]. In turn, Robo activates the Dpp/TGF-beta signaling pathway, which suppresses the Myc TF expression and PSC cell proliferation (*Fig. 2*) [33, 123, 126].

Another important discovery has been that outside signals, namely from the nervous and humoral systems, directly affect the state and function of PSC cells. Insulin-like peptides expressed in neurons, glia, and fat body cells [153] regulate the proliferation and growth of PSC cells through insulin signaling pathway activation [31, 32, 104, 122, 154]. Inhibition of this pathway through inactivation of its various components such as the insulin receptor (InR), Akt1, phosphoinositide-dependent kinase 1 (Pdk1), and phosphoinositide-3-kinase (PI3K) reduces the number of PSC cells. It has also been discovered that activation of the rapamycin signaling pathway is involved in this process. Further studies will investigate the interactions between the detected signaling pathways and their role in the regulation of maintenance and functioning of the cells of the hematopoietic niche, which is central in regulating hematopoiesis in *Drosophila*’s hematopoietic organ.

## CONCLUSIONS

Over the past 20 years, significant progress has been made in our understanding of the molecular mechanisms regulating hematopoiesis in the fruit fly. As the most genetically advanced model system, *Drosophila *has allowed us to describe the complex interactions between signaling pathways and the TFs involved in the regulation of the maintenance and differentiation of multipotent hemocyte precursor cells, namely preprohemocytes and prohemocytes. These cells differentiate during larval development into three types of mature hemocytes: plasmatocytes, crystal cells, and lamellocytes. It has been shown that in insects, as in mammals, the main role in the maintenance and regulation of the differentiation of hematopoietic progenitor cells is played by hematopoietic niche cells – PSC cells. Determination of these cells’ fate occurs in parallel with the specification of hematopoietic progenitors in the HO. Throughout the larval instar, PSC cells coordinate prohemocyte maintenance and differentiation through secreted ligands (Hh, Pvf1, Ser, Wg/Wnt), activating the appropriate signaling pathways in hemocyte precursors. These signals are involved, among others, in the maintenance of the autocrine and paracrine signals (Wnt/ beta-catenin, calcium signaling, AFC, Stat92E) in prohemocytes, activating or inhibiting their maintenance in an undifferentiated state. Prohemocytes are maintained in a mitotically quiescent state in the MZ of the hemopoietic organ. In addition, a two-way equilibrium regulation of prohemocyte maintenance has been proven to take place through signals from differentiated (Pvr, Adgf-A, AdoR, PKA) and PSC cells (Hh, Pvf1). Recent studies using single-cell transcriptome sequencing have shown the presence of intermediate stages of prohemocyte differentiation and uncharacterized populations of mature hemocytes. Prohemocyte differentiation occurs in the so-called intermediate zone, where cells begin to divide and become susceptible to differentiation signals. But this mechanism requires further investigation. In addition, recent studies have shown that DV cells also serve as a type of hematopoietic niche, participating in prohemocyte maintenance. To date, HSCs capable of self-renewal by asymmetric cell division have not been identified in *Drosophila. *However, the most naive preprohemocyte population has been identified. These cells are regulated by PSC cells via the activation of the Notch, Dpp, and Pvf2/Pvr signaling pathways. In addition to maintaining hemocyte precursors, PSC cells participate in the regulation of the cellular immune response and the cells mediating melanization and inactivation of pathogenic objects through the Spi/EGFR, Toll, and Ser/Notch signaling pathways. Based on the results of the reviewed studies, a unique picture of the interaction of the molecular mechanisms regulating hematopoiesis in one of the representatives of arthropods has emerged. The genetic model of *Drosophila *has allowed us to decipher the molecular events that regulate hematopoiesisin in greater detail, and in some aspects, has proven to be ahead of the murine model.

## Abbreviations

HO: hematopoietic organ (lymph glands)
DC: dorsal vessel
PPHs: preprohemocytes
PHs: prohemocytes
IPHs: intermediate prohemocytes
PSC: posterior signaling center
MZ: medullary zone
CZ: cortical zone
IZ: intermediate zone
PL: plasmatocytes
CC: crystalline cells
LM: lamellocytes
ROS: reactive oxygen species
AMP: antimicrobial peptides
ECM: extracellular matrix
UAS: upstream activation sequence
scRNAseq: single-cell RNA sequencing
HSCs: hematopoietic stem cells
SCs: stem cells
AGM: aorta-gonad-mesonephros
Odd: Odd-skiped
Crq: Croquemort
TF: transcription factor
Antp: Antennapedia
NimC1: Nimrod C1
Col: Collier
Hth: Homothorax
Tin: Tinman
Pnr: Pannier
FGFR: fibroblast growth factor receptor
Htl: Heartless
Dpp: Decapentaplegic
Wg: Wingless
Hh: Hedgehog
Ser: Serrate
Dome: Domeless
E-cad: E-cadherin
Vkg: Viking
Hml: Hemollectin
PPO: prophenoloxidase
Lz: Lozenge
Hnt: Hindsight
Fz: Frizzled
Dot: Dorothy
Mad: mothers against dpp
Sd: Scalloped
Ptc: Patched
Ci: Cubitus interruptus
EGFR: epidermal growth factor receptor
TGF-beta: transforming growth factor beta
PCP: planar cell polarity
FGF: fibroblast growth factor
Upd1-3: Unpaired 1-3
FoxO: forkhead box protein O
Adgf-A: adenosine deaminase growth factor-A
AdoR: adenosine receptor
PKA: protein kinase A
